# Relationships between cerebral small vessel diseases markers and cognitive performance in stroke-free patients with atrial fibrillation

**DOI:** 10.3389/fnagi.2022.1045910

**Published:** 2023-01-04

**Authors:** Wendan Tao, Junfeng Liu, Chen Ye, William Robert Kwapong, Anmo Wang, Zhetao Wang, Shi Chen, Ming Liu

**Affiliations:** ^1^Department of Neurology, West China Hospital, Sichuan University, Chengdu, China; ^2^Department of Radiology, West China Hospital, Sichuan University, Chengdu, China; ^3^Department of Cardiology, West China Hospital, Sichuan University, Chengdu, China

**Keywords:** atrial fibrillation, cognitive decline, cerebral small vessel disease, magnetic resonance imaging, brain, stroke-free

## Abstract

**Background:**

Atrial fibrillation (AF) is related to an increased risk of cognitive dysfunction. Besides clinically overt stroke, AF can damage the brain *via* several pathophysiological mechanisms. We aimed to assess the potential mediating role of cerebral small vessel disease (SVD) and cognitive performance in individuals with AF.

**Methods:**

Stroke-free individuals with AF from the cardiological outpatient clinic at West China Hospital of Sichuan University were recruited. Extensive neuropsychological testing tools were assessed including global function, domains of attention, executive functions, learning, and memory. 3 T magnetic resonance imaging (MRI) was used for SVD markers assessment of white matter hyperintensities (WMH), lacunes, cerebral microbleeds (CMBs), and enlarged perivascular spaces (EPVS). The correlation between SVD markers and cognitive measures was analyzed by multivariate linear regression models.

**Results:**

We finally enrolled 158 participants, of whom 95 (60.1%) were males. In multivariate models, the presence of lacunes independently associated with Montreal Cognitive Assessment (Model 1: *ß* = 0.52, Model 2: *ß* = 0.55), Rey Auditory Verbal Learning Test-immediate and delayed recall (Model 1: *ß* = 0.49; *ß* = 0.69; Model 2: *ß* = 0.53; *ß* = 0.73) as well as Stroop-A_correct_ (Model 1: *ß* = 0.12; Model 2: *ß* = 0.13), while total WMH severity independently associated with Stroop_time_-A (Model 1: *ß* = 0.24; Model 3: *ß* = 0.27), Stroop_time_-B (Model 1: *ß* = 0.17; Model 3: *ß* = 0.17), Stroop_time_-C (Model 1: *ß* = 0.22; Model 3: *ß* = 0.21) and Shape Trail Test-A (Model 1: *ß* = 0.17; Model 3: *ß* = 0.16).

**Conclusion:**

In our cohort of stroke-free individuals with AF, lacunes, and WMHs were independently associated with cognitive decline while EPVS and CMBs did not show significance. Assessment of SVD MRI markers might be valuable for cognition risk stratification and facilitate optimal management of patients with AF.

## Introduction

Atrial fibrillation (AF) is the commonest sustained arrhythmia, and it is suggested to increase in the coming years (Hindricks et al., 2021). AF is increasingly recognized as an important risk factor for cognitive impairment (Ihara and Washida, 2018). AF increases the risk of stroke, and stroke was considered a major cause for significantly faster cognitive decline and dementia in such patients (Mead and Keir, 2001). However, numerous reports observed that AF is associated with cognitive impairment and dementia independent of clinically overt stroke (Olson and Grose, 1997; Knecht et al., 2008; Marzona et al., 2012; Stefansdottir et al., 2013; Chen et al., 2014; de Bruijn et al., 2015; Ihara and Washida, 2018).

Recently, evidence observed preexisting cognitive impairment in patients with AF–associated ischemic stroke or transient ischemic attack is associated with neuroimaging markers of cerebral small vessel disease (SVD; Banerjee et al., 2020). SVD is diagnosed on conventional magnetic resonance imaging (MRI) as lacunes, enlarged perivascular spaces (EPVS), cerebral microbleeds (CMBs), and white matter hyperintensities (WMHs). These radiological markers are suggested to reflect the cerebral microvasculature and are linked to cognitive decline (Jokinen et al., 2020). It is suggested that multiple mechanisms were involved in increasing the burden of SVD in patients with AF, such as hemodynamic instability, microembolism, inflammation, endothelial dysfunction, and lymphatic system dysfunction (Kobayashi et al., 2012; Horstmann et al., 2015; Wiggins et al., 2020; Jin et al., 2022). In recent studies, SVD biomarkers have demonstrated utility in distinguishing between vascular and neurodegenerative (mainly Alzheimer’s disease-related) cognitive impairment (Palesi et al., 2018). However, these imaging markers have different pathological backgrounds affecting cognitive performance variably. While previous studies showed clues regarding the association between SVD markers and AF patients (Ding et al., 2021; Du et al., 2021), the potential mediating role of SVD MRI markers for cognitive performance in patients with AF is largely unknown. Assessment of SVD markers might be valuable for cognition risk stratification and facilitate optimal management for patients with AF.

In our present study, we aim to explore whether there are independent associations between SVD markers and cognitive impairment; we adopted comprehensive neurocognitive evaluation in a cohort of stroke-free individuals with AF.

## Materials and methods

### Participants

Patients with AF were prospectively recruited from March 2021 to May 2022 in the cardiology clinic of West China Hospital, Sichuan University. The diagnosis of AF is based on a rhythm record with an electrocardiography (ECG) tracing showing AF. Paroxysmal or persistent/permanent AF was classified according to 2020 European Society of Cardiology guidelines (Hindricks et al., 2021). The final diagnosis and subtype of AF were verified by a cardiologist. Subjects who refused or have contraindications to an MRI scan or could not cooperate with neuropsychological evaluation were excluded. To reduce the possible effects of stroke on cognitive function, we restricted this study to patients without a history or clinical evidence of stroke or asymptomatic cortical or subcortical non-lacunar stroke. Patients with coexisting central nervous system diseases affecting cognitive function (e.g., Alzheimer’s disease, cerebral amyloid angiopathy, etc.) were excluded. The neurological function was evaluated by a neurologist. Patients with other severe neurological conditions or treated with psychotropic medication were also excluded.

All patients received a clinical face-to-face interview and baseline characteristics including age, sex, years of education, and vascular risk factors (diabetes mellitus, hyperlipidemia, hypertension, coronary artery disease, current alcohol consumption, and current smoking) were systematically recorded. CHA2DS2-VASc score (congestive heart failure, hypertension, age ≥75 years, diabetes mellitus, stroke or transient ischemic attack (TIA), vascular disease, age 65 to 74 years, sex category) assessing for stroke risk categorization was recorded (Lip et al., 2010). Body mass index (BMI) was calculated from measured height and weight (Boriani et al., 2019). The protocol of our study was approved by the Medical Ethics Committee of West China Hospital, Sichuan University (2020–104), based on the principles in the “Declaration of Helsinki.” Written informed consent was obtained from all participants.

### Neuropsychological assessment

Trained research technicians supervised by a clinical neuropsychologist conducted a battery of neuropsychological tests. The cognitive test battery comprised of Mini-Mental State Examination (MMSE), Montreal Cognitive Assessment-Beijing version (MoCA-BJ), Chinese Rey Auditory Verbal Learning Test (C-RAVLT), Stroop’s color word test (SCWT), and Shape Trail Test (STT). The MMSE and MoCA-BJ were used to assess global cognition (Huang et al., 2021).

The Chinese Rey Auditory Verbal Learning Test (C-RAVLT) was developed to rate immediate and delayed episodic verbal memory (Boone et al., 2005). There are five successive presentations of the original list of 15 words (List A) in the test, and with every trial followed by a free recall. The sum of words recalled on the 5 trials was taken as the score for immediate recall (IR). After a 30-min delay, subjects were requested to recall words from List A again and the number of words recalled was taken as delayed recall (DR).

For Shape Trail Test (STT), patients underwent STT-A (drawing a line between 25 consecutive numbers as fast as possible) and STT-B (linking numbers alternating between circles and squares; Zhao et al., 2013). Times for completing each part of the task was recorded.

Stroop Color and Word Test (SCWT) consists of three cards printed in color (Zysset et al., 2001). Card A is the word subtask which consists of common words unrelated to the concept of colors, card B is the dot subtask that consists of color dots, and card C is the color-word subtask that consists of words in color. Blue, green, red, and yellow are the colors used. Subjects were required to name the colors in which the stimuli were printed and to disregard their verbal content. For the Chinese version of the SCWT, common Chinese characters unrelated to the concept of color were selected. Scores are derived from the completion times and correct numbers in each part of the subtasks.

### Imaging acquisition and volumetric measures of brain structure

Brain imaging was performed using a standard 3 T MRI scanner (Siemens Skyra) with a 32-channel head coil at West China Hospital of Sichuan University. The scanning sequences and imaging parameters were the same as those we reported in our previous study (Tao et al., 2022). T1-weighted structural imaging was processed using Computational Anatomy Toolbox 12 (CAT12) for Statistical Parametric Mapping (SPM) 12 (Wellcome Trust Center for Neuroimaging, London, UK). Postprocessing of brain structure measures was reported in our previous study (Tao et al., 2022). Voxel-based Morphometry (VBM) was used as comparing the absolute volume of gray or white matter structures. Total intracranial volumes (TIVs) consist of gray matter volumes (GMV), white matter volumes (WMV), and cerebrospinal fluid. Automated anatomical labeling (AAL) template was used to calculate bilateral hippocampus volumes (HV).

### SVD neuroimaging markers rating

SVD MRI markers were rated according to the STandards for ReportIng Vascular changes on nEuroimaging (STRIVE)consensus criteria (Wardlaw et al., 2013). Evaluation of lacunes, white matter hyperintensity (WMH), cerebral microbleeds (CMBs), and enlarged perivascular spaces (EPVS) is well detailed in our previous report (Tao et al., 2022). Fazekas scale (0–3) was used to rate the extent of periventricular and deep WMH. The total WMH score was defined as the sum of the scores for deep and periventricular WMH. Numbers of EPVS were measured separately in the basal ganglia (EPVS-BG) and centrum semiovale (EPVS-CSO) and classified to a 3-category ordinal scale (0–10, 10–25, and >25).

An experienced doctor (YC) who was blinded to the clinical information of the participants used software (RadiAnt DICOM Viewer1.0.4.4439; Medixant Ltd., Poznan, Poland) to visually read the SVD MRI markers. A random sample of 30 patients was selected and evaluated by a second rater (TWD). There was a good inter-rater agreement for the total WMH severity (kappa 0.78, *p* < 0.001), EPVS-CSO (kappa 0.70, *p* < 0.001), EPVS-BG (kappa 0.80, *p* < 0.001) and the presence of lacunes (kappa 0.85, *p* < 0.001).

## Statistical analysis

Continuous variables were presented as mean ± standard deviation (SD) or median and interquartile ranges while categorical variables were expressed as frequencies and percentages. All cognitive tests and brain structure measures were standardized as the z scores, calculated by subtracting the mean value from the value of the observation and dividing by the standard deviation. Pearson’s or point-biserial r_pb_ correlation of univariate analyses was applied to test the influence of demographics, AF features, vascular risk factors as well as SVD markers on cognitive function. Independent multivariate linear regression based on generalized linear models was used to examine the relationship between cognitive trials as the dependent variable, and SVD markers as the main independent variables. G Power software was used for power analysis (Faul et al., 2009). It is indicated that we need at least 153 participants to detect the effect (actual power = 0.95) with parameters (Effect size f2 = 0.15, α = 0.05, 1-β = 0.95, number of predictors = 7) to conduct a linear multiple regression. In model 1, variables in baseline with *p* < 0.05 in univariate analysis were adjusted. Confounders such as education years and brain volumes associated with cognitive performance were adjusted as well. In model 2, total WMH score were additionally adjusted. In model 3, the presence of lacunes were additionally adjusted. In addition, subgroup analyses were performed using stratified logistic regression models. The significance of interaction (*p* for interaction) was tested using the likelihood ratio test. Statistical analyses were performed with SPSS (version 24, SPSS Inc.), with *p* < 0.05 being set as statistically significant.

## Results

We initially enrolled 189 neurologically normal patients with AF. Out of the participants, 5 patients refused or had a contradiction to MR imaging, 16 patients had an asymptomatic embolic stroke or history of stroke, 8 patients did not complete clinical evaluation and 2 patients had possible cerebral amyloid angiopathy.

Our final data analysis included 158 patients who had their MRI scans as well as cognitive assessment. The mean age was 63.6 ± 9.6 years and 60.1% (n = 95) were males. The mean BMI was 24.8 ± 3.7 kg/m^2^ and 44.9% (n = 71) had persistent/permanent AF. Median CHA_2_DS_2_-VASc score was 2 (interquartile range, IQR 1–3). Eighty-seven (55.1%) patients were on anticoagulation drugs while 14 (8.9%) patients were on antiplatelet drugs. The median MoCA score was 25 (IQR, 22–27) and the MMSE score was 28 (IQR, 26–30). Information on vascular risk factors and MRI characteristics is shown in Table 1.

**Table 1:** Baseline characteristics of the study cohort (*n* = 158).

Characteristics	Value
Age, y	63.6 ± 9.6
Male sex	95 (60.1)
BMI (kg/m^2^)	24.8 ± 3.7
Persistent /permanent AF	71 (44.9)
Education years	9 (9–12)
Vascular risk factors	
Hypertension	58 (36.7)
Diabetes mellitus	24 (15.2)
Hyperlipidemia	27 (17.1)
Coronary heart disease	16 (10.1)
Current smoking	49 (31.0)
Current drinking	51 (32.3)
CHA_2_DS_2_-VASc score	2.0 (1.0–3.0)
SVD imaging markers	
**Lacunes**	
Presence of Lacunes	19 (12.0)
lacunes numbers,range	1–7
**CMBs**	
Presence of CMBs	36 (22.7)
Strictly Lobar CMB	15 (9.5)
Deep CMB^a^	21 (13.3)
numbers range	1–9
**EPVS**	
Severity of BG-EPVS	1.0 (1.0–2.0)
Severity of CSO-EPVS	1.0 (1.0–2.0)
**WMH**	
pWMH	1.0 (1.0–1.0)
dWMH	1.0 (1.0–2.0)
Total WMH score	2.0 (2.0–3.0)
TIV	1,442.8 (1318.2–1545.7)
Average HV	6.9 (6.4–7.4)
GMV	591.3 (554.5–620.6)
WMV	489.2 (446.7–530.9)
Medication	
Anticoagulation	87 (55.1)
Antiplatelet	14 (8.9)

Data are *n* (%), mean (SD) or median (IQR).

a With or without lobar CMB.

BMI: body mass index; AF: atrial fibrillation; CHA2DS2-VASc (congestive heart failure, hypertension, age ≥ 75 years, diabetes mellitus, stroke or transient ischemic attack (TIA), vascular disease, age 65 to 74 years, sex category); SVD: cerebral small vessel disease; CMBs: cerebral microbleeds; EPVS: enlarged perivascular spaces; BG: basal ganglia; CSO: centrum semiovale; pWMH: periventricular white matter hyperintensity; dWMH: deep white matter hyperintensity; TIV: Total Intracranial Volume; HV: Hippocampal Volume; GMV: Gray Matter Volume; WMV: White Matter Volume.

We assessed the influence of demographics on cognitive function. Older age significantly correlated with lower correction number in memory (RAVLT-IR, *r* = −0.18, *p* = 0.033; RAVLT-DR, *r* = −0.23, *p* = 0.005), longer completion time for Stroop test (part A, *r* = 0.18, *p* = 0.029; part B, *r* = 0.26, *p* = 0.002; part C, *r* = 0.21, *p* = 0.012) and STT (part A, *r* = 0.23, *p* = 0.015; part B, *r* = 0.34, *p* < 0.001). Compared to females, males had a significantly better performance in MoCA (*r*_pb_ = 0.28, *p* < 0.001) and MMSE (*r*_pb_ = 0.22, *p* = 0.004; Supplementary Table 1). Therefore, we introduced age and sex as independent variables in multivariate analysis.

Concerning the effect of AF features and vascular risk factors on cognitive function, we find the presence of hypertension correlated with a longer completion time of STT-B (*r*_pb_ = −0.19, *p* = 0.042; Supplementary Table 2). However, there is no significant correlation between CHA2DS2-VASc score, persistent AF, anticoagulation, or antiplatelet treatment, and cognitive performance. Thus, hypertension was considered an independent variable in multivariate analysis.

Table 2 shows the univariate associations between SVD markers and cognitive performance. The presence of lacunes significantly correlated with MoCA (*r* = −0.19), tasks of learning and memory (RAVLT-IR, *r* = −0.21; RAVLT-DR, *r* = −0.27) as well as attention and executive functions (Stroop-A_correct_, *r* = −0.23). The total WMH severity significantly correlated with attention and executive function (Stroop_time_-A, *r* = 0.34; Stroop_time_-B, *r* = 0.27; Stroop_time_-C, *r* = 0.25; STT-A, *r* = 0.25; STT-B, *r* = 0.24). CMBs and EPVS did not correlate with any cognitive tests.

**Table 2:** Univariate analysis between SVD imaging markers and standardized cognitive measures.

Variables	The presence of CMBs		The presence of lacunes		Total WMH		BG-EPVS		CSO-EPVS	
	*r*	*p* value	*r*	*p* value	*r*	*p* value	*r*	*p* value	*r*	*p* value
MMSE	0.10	0.201	−0.05	0.503	−0.09	0.259	−0.06	0.491	0.02	0.773
MoCA-BJ	0.05	0.475	**−0.19**	**0.018**	−0.05	0.561	−0.12	0.129	0.004	0.962
C-RAVLT (IR)	−0.01	0.863	**−0.21**	**0.012**	−0.04	0.629	−0.07	0.436	0.013	0.873
C-RAVLT (DR)	0.008	0.920	**−0.27**	**0.001**	−0.07	0.391	−0.11	0.197	−0.08	0.330
Stroop-A_time_,s	−0.04	0.586	0.04	0.654	**0.34**	**<0.001**	0.04	0.607	0.11	0.215
Stroop-A_correct_	−0.008	0.923	**−0.23**	**0.006**	−0.02	0.833	−0.04	0.615	−0.09	0.262
Stroop-B_time_,s	0.06	0.512	0.06	0.469	**0.27**	**0.001**	−0.07	0.431	0.15	0.071
Stroop-B_correct_	0.06	0.467	0.03	0.731	−0.04	0.585	0.06	0.503	0.07	0.414
Stroop-C_time_,s	0.09	0.239	0.12	0.150	**0.25**	**0.002**	0.02	0.819	0.14	0.102
Stroop-C_correct_	−0.06	0.496	−0.10	0.217	−0.16	0.059	−0.02	0.799	−0.11	0.198
STT-A,s	−0.05	0.624	0.08	0.338	**0.25**	**0.006**	0.10	0.302	0.10	0.298
STT-B,s	−0.09	0.297	0.08	0.419	**0.24**	**0.010**	0.02	0.806	0.03	0.785

SVD, cerebral small vessel disease; CMB, cerebral microbleeds; WMH, white matter hyperintensity; EPVS, enlarged perivascular spaces; BG: basal ganglia; CSO, centrum semiovale; MMSE, Mini-Mental State Examination; MoCA, Montreal Cognitive Assessment-Beijing version; C-RAVLT (IR), The Chinese Rey Auditory Verbal Learning Test (immediate recall); C-RAVLT (DR), The Chinese Rey Auditory Verbal Learning Test (delayed recall); STT, Shape Trail Test. Bold values are statistically significant.

**Figure 1:**
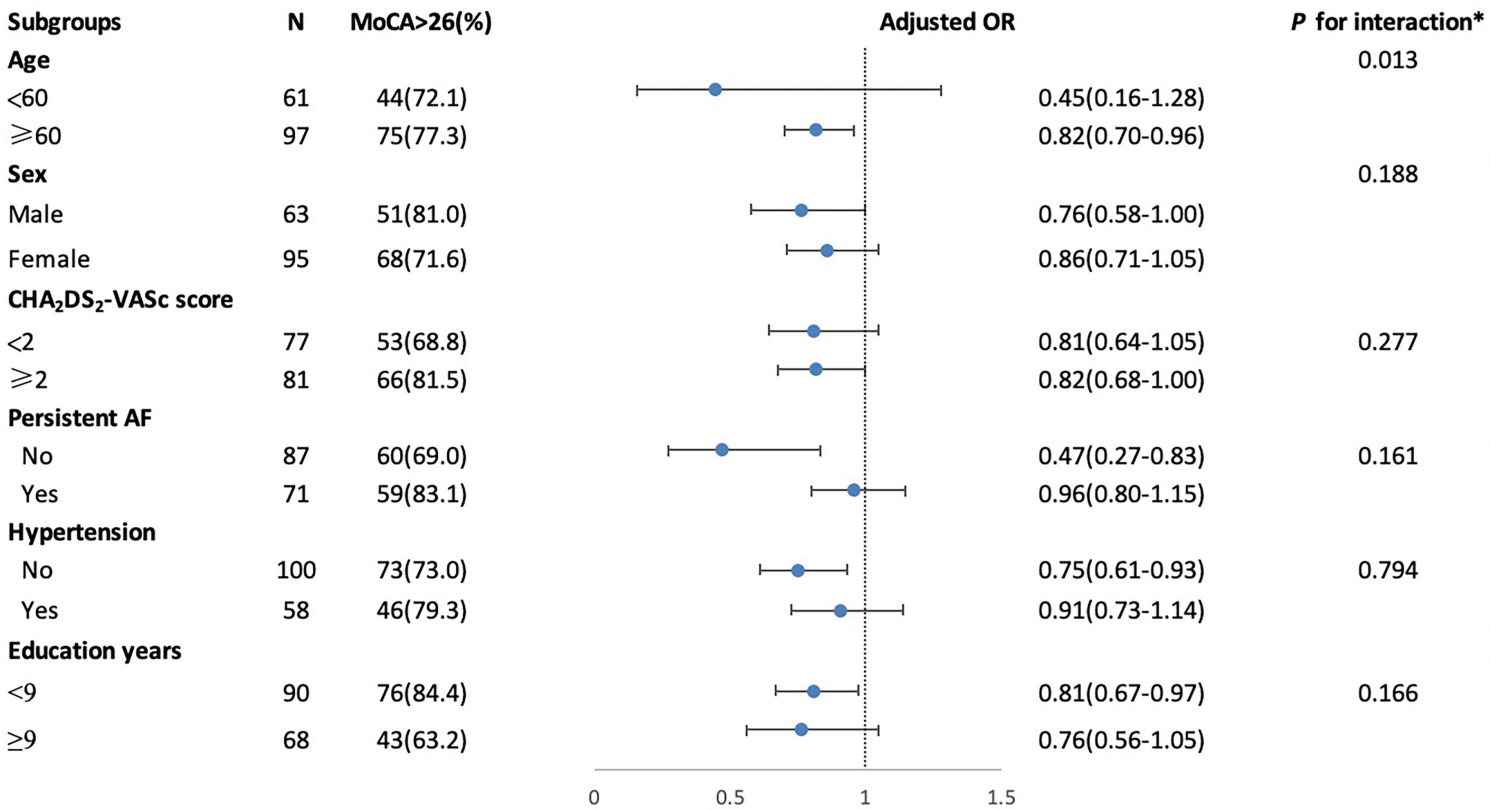
Stratified logistic regression analysis to identify variables modify the correlation between the presence of lacunes and MoCA. *Above model adjusted for age, sex, education years, hypertension, CHA2DS2-VASc score (congestive heart failure, hypertension, age ≥ 75 years, diabetes mellitus, stroke or transient ischemic attack (TIA), vascular disease, age 65 to 74 years, sex category) and persistent AF (atrial fibrillation). In each case, the model is not adjusted for the stratification variable.

In Table 3, after adjusting for age, sex, education years, hypertension, and brain volumes (gray matter volumes, white matter volumes, and average hippocampal volume) in model 1, or additionally adjusted for total WMH severity in model 2, the presence of lacunes showed a significant association with MoCA (Model 1: *ß* = 0.52, Model 2: *ß* = 0.55) and RAVLT-immediate and delayed recall (Model 1: *ß* = 0.49; *ß* = 0.69; Model 2: *ß* = 0.53; *ß* = 0.73) as well as Stroop-A_correct_ (Model 1: *ß* = 0.12; Model 2: *ß* = 0.13). After adjusted in model 1 or additionally adjusted for the presence of lacunes in model 3, total WMH severity is still independently associated with Stroop_time_-A (Model 1: *ß* = 0.24; Model 3: *ß* = 0.27), Stroop_time_-B (Model 1: *ß* = 0.17; Model 3: *ß* = 0.17), Stroop_time_-C (Model 1: *ß* = 0.22; Model 3: *ß* = 0.21) and STT-A (Model 1: *ß* = 0.17; Model 3: *ß* = 0.16). Since only lacunes showed an independent association with global cognitive function, we did a stratified analysis to identify variables that may modify the association between the presence of lacunes and MoCA in Figure 1. We found that the relationship between the presence of lacunes and MoCA was changed by age (*p* for interaction = 0.013), but did not change by sex, hypertension, persistent AF, CHA_2_DS_2_-VASc score, or education years.

**Table 3:** Correlation between SVD imaging markers and standardized cognitive measures in generalized linear models.

Variables		The presence of lacunes				Total WMH severity			
		Model 1		Model 2		Model 1		Model 3	
	Median (IQR)	*ß* coefficient (95% CI)^a^	*p* value	*ß* coefficient (95% CI)^b^	*p* value	*ß* coefficient (95% CI)^a^	*p* value	*ß* coefficient (95% CI)^c^	*p* value
MMSE	28 (26–30)	0.08 (−0.34to 0.51)	0.711	0.08 (−0.35to 0.51)	0.722	−0.008 (−0.14 to 0.12)	0.902	−0.004 (−0.14 to 0.13)	0.950
MoCA	25 (22–27)	**0.52 (0.14 to 0.90)**	**0.008**	**0.55 (0.16 to 0.94)**	**0.005**	0.03 (−0.09 to 0.15)	0.652	0.06 (−0.06 to 0.18)	0.358
RAVLT (IR)	35 (27–45)	**0.49 (0.06 to 0.94)**	**0.027**	**0.53 (0.09 to 0.97)**	**0.020**	0.04 (−0.10 to 0.19)	0.552	0.07 (−0.08 to 0.22)	0.343
RAVLT (DR)	8 (5–10)	**0.69 (0.24 to 1.14)**	**0.003**	**0.73 (0.27 to 1.18)**	**0.002**	0.06 (−0.09 to 0.21)	0.476	0.09 (−0.06 to 0.24)	0.240
Stroop-A_time_,s	32 (27–40)	0.02 (−0.44 to 0.47)	0.946	0.17 (−0.27to 0.61)	0.460	**0.24 (0.10 to 0.38)**	**<0.001**	**0.27 (0.13 to 0.42)**	**<0.001**
Stroop-A_correct_	50 (50–50)	**0.12 (0.02 to 0.22)**	**0.015**	**0.13 (0.03 to 0.23)**	**0.009**	0.01 (−0.02 to 0.04)	0.471	0.02 (−0.01 to 0.05)	0.229
Stroop-B_time_,s	55 (44–65)	0.06 (−0.43 to 0.56)	0.815	0.17 (−0.33 to 0.66)	0.516	**0.17 (0.01 to 0.33)**	**0.043**	**0.17 (0.01 to 0.34)**	**0.034**
Stroop-B_correct_	50 (48–50)	−0.22 (−0.77 to 0.33)	0.435	−0.20 (−0.76 to 0.36)	0.481	0.04 (−0.14 to 0.23)	0.645	0.03 (−0.16 to 0.22)	0.753
Stroop-C_time_,s	93 (74–123)	−0.19 (−0.72 to 0.32)	0.464	−0.07 (−0.59 to 0.45)	0.800	**0.22 (0.05 to 0.39)**	**0.011**	**0.21 (0.04 to 0.38)**	**0.015**
Stroop-C_correct_	49 (45–50)	0.18 (−0.25 to 0.62)	0.417	0.13 (−0.31 to 0.57)	0.551	−0.08 (−0.22 to 0.06)	0.246	−0.08 (−0.22 to 0.07)	0.308
STT-A,s	30 (20–52)	−0.33 (−0.85 to 0.19)	0.211	−0.30 (−0.81 to 0.21)	0.246	**0.17 (−0.007 to 0.33)**	**0.041**	**0.16 (0.03 to 0.32)**	**0.046**
STT-B,s	175 (129–228)	−0.31 (−0.71 to 0.09)	0.126	−0.30 (−0.68 to 0.10)	0.141	0.08 (−0.05 to 0.21)	0.237	0.08 (−0.05 to 0.20)	0.235

SVD, cerebral small vessel disease; CMB, cerebral microbleeds; WMH, white matter hyperintensity; EPVS, enlarged perivascular spaces; BG: basal ganglia; CSO, centrum semiovale; MMSE, Mini-Mental State Examination; MoCA, Montreal Cognitive Assessment-Beijing version; C-RAVLT (IR), The Chinese Rey Auditory Verbal Learning Test (immediate recall); C-RAVLT (DR), The Chinese Rey Auditory Verbal Learning Test (delayed recall); STT, Shape Trail Test. Bold values are statistically significant.

a Model 1: adjusted for age, sex, hypertension, education years, average hippocampal volume; gray matter volume; white matter volume.

b Model 2: additionally adjusted for total WMH severity.

c Model 3: additonally adjusted for the presence of lacunes.

## Discussion

AF commonly coexists with other etiologies, including SVD^18^. Thus far, the potential mediating role of SVD for cognitive decline in patients with AF is unclear. In the present cohort study of stroke-free individuals with AF, we demonstrated that lacunes and WMHs were independently associated while EPVS and microbleeds had weaker associations with cognitive decline. Compared to previous studies on cognition in AF, patients recruited in our study were younger (Stefansdottir et al., 2013; Bonnesen et al., 2021) thus age-related changes on the brain could have little impact on their cognition.

Our results showed the presence of lacunes was related to both global and domain-specific cognitive performance changes in patients with AF. These findings remained consistent after additionally adjusting the impact of WMH. Few studies report the association between lacunes and cognitive performance in patients with AF. Previous studies found increased odds of subclinical cerebral infarctions (SCIs) in patients with AF which might contribute to cognitive decline (Chen et al., 2014; Conen et al., 2019). They demonstrated that SCIs were possibly caused by cardiogenic embolism or *in-situ* small vessel disease, however, most of them did not report the proportion of lacunes or the location (cortical or subcortical, or deep) or sizes of these lesions. Lately, data from the CROMIS-2 (Clinical Relevance of Microbleeds in Stroke 2) study indicated lacunes were associated with preexisting cognitive impairment in patients with AF-related cardioembolic stroke or transient ischemic attacks (Banerjee et al., 2020). We hypothesized that the disturbed cerebral perfusion, embolism or more common vascular comorbidities (e.g., hypertension, diabetes, and smoking) in patients with AF exert a synergistic effect on the formation of lacunes. We showed that the presence of lacunes correlated with worse performance in tasks of global function, learning and memory, attention and executive function. In a multinational Leukoaraiosis and Disability (LADIS) study, the authors showed that incident lacunes on MRI images led to the deterioration of the executive and psychomotor speed (Jokinen et al., 2011), although the presence of lacunes (one or two) contributing to cognitive decline is often considered negligible (Fisher, 1982). In a healthy older adult cohort, lacunar volumes significantly correlated with global cognitive function, cognitive processes, and executive function (Jokinen et al., 2020); In our AF cohort, we found that patients presenting with lacunes had worse memory performance. Memory impairment is usually the first sign that a person may be developing cognitive impairment. Whether lacunes in AF patients could be regarded as an early imaging sign of cognitive decline is needed to be verified by large prospective cohort studies.

We also found that WMH was significantly associated with executive functions and psychomotor speed in patients with AF. Increased WMH lesion burden among patients with AF was reported by several studies (Henon et al., 1996; de Leeuw et al., 2000; Bernstein et al., 2015). In several longitudinal studies, subjects with AF have been observed with severe periventricular white matter lesions or accelerated progression of white matter lesions, independent of cerebral infarcts (de Leeuw et al., 2000; Ding et al., 2021). But a previous study did not find prevalent AF was independently associated with white matter disease, using white matter microstructural integrity and WMH volume as markers (Shao et al., 2019). In a moderate-size of cohort, it was observed that AF with embolic stroke mechanism was significantly associated with specific WMH lesion patterns, presented as anterior subcortical WMH patches (Mayasi et al., 2018). AF-preferred anterior WMH topography is associated with a thinner frontal cortex and cognitive decline (Seo et al., 2012). Therefore, the identification of the WMH progression and distribution may help us better understand the clinical cognitive decline observed among patients with AF.

Our data did not demonstrate that CMBs are related to cognitive performance in subjects with AF. CMBs were detected in 23.8% of subjects with AF in our study, which was similar to previous studies (Horstmann et al., 2015; Haji et al., 2016). We observed the percentage of strictly lobar CMBs was lower than deep CMB (strictly lobar, 9.4% vs. deep, 14.4%). The prevalence and location of CMBs in non-stroke cohorts with AF are little known, although previous evidence suggested that CMBs may be more common in lobar than in deep areas in patients with AF-associated ischemic stroke (Song et al., 2014; Selim and Diener, 2017). The Rotterdam study revealed an association between lobar CMBs and a decline in executive functions, memory, and processing speed, whereas CMBs in other brain regions was related to a decline in processing and motor speed; however, the study was based on the general population rather than subjects with AF (Haji et al., 2016). There is little pathological evidence to verify the underlying pathogenesis of CMBs in AF patients. Thus, CMBs in patients with atrial fibrillation might commingle with other conditions, such as white matter disease, antithrombotic use, or many classical risk factors for cerebrovascular disease (Romero et al., 2014; Selim and Diener, 2017). Since the moderate sample size of our study for CMB detection, a large sample cohort of AF is needed to ascertain the prognostic significance of CMBs for cognitive functions and to develop optimal management strategies.

There were limitations to our study. A major limitation of our study is the lack of detailed assessment of the Alzheimer’s disease pathology in our study participants. Accumulating reports have shown that AF is a risk factor for Alzheimer’s disease (Ihara and Washida, 2018; Islam et al., 2019), and our current study did not perform a detailed examination to exclude participants with probable AD or diagnosed AD. Future studies should incorporate the use of positron emission tomography (PET), cerebral spinal fluid (CSF) biomarkers of phosphorylated tau and Aβ in addition to clinical dementia rating (CDR) to exclude probable AD or diagnosed AD in the AF cohort. Furthermore, the number of participants with AF was relatively small. Most of our included patients were those consulting for radio-frequency catheter ablation in the cardiology outpatient department, therefore, there is inevitable selection bias since AF patients with severe heart failure were not recruited. The number of patients receiving anticoagulation was relatively low, suggesting a potential selection bias. Another potential shortcoming is the cross-sectional design, which could not assess the causality or directionality of the effect.

## Conclusion

In this cohort of stroke-free patients with AF, our analyses show that lacunes and white matter hyperintensity have an impact on cognitive function. Conversely, microbleeds and EPVS were not significantly associated with cognitive decline. Assessment of SVD MRI markers in patients with AF might be valuable for cognition risk stratification and facilitate optimal management of AF patients.

## Data availability statement

The raw data supporting the conclusions of this article will be made available by the authors, without undue reservation.

## Ethics statement

The studies involving human participants were reviewed and approved by the Medical Ethics Committee of West China Hospital, Sichuan University. The patients/participants provided their written informed consent to participate in this study.

## Author contributions

WT and JL: equally contributed to this study. WT: study concept, data statistical analysis, and writing the paper. JL: study protocol design and data analysis. WK: paper revision. CY and AW: patient recruitment and imaging data analysis: ZW: imaging data scanning. SC: patient recruitment and study protocol development. ML: study concept and guidance. All authors contributed to the article and approved the submitted version.

## Funding

This work was funded by The National Natural Science Foundation of China (Grant No. 81601022), the 1.3.5 project for disciplines of excellence, West China Hospital, Sichuan University (ZYGD18009).

## Conflict of interest

The authors declare that the research was conducted in the absence of any commercial or financial relationships that could be construed as a potential conflict of interest.

## Publisher’s note

All claims expressed in this article are solely those of the authors and do not necessarily represent those of their affiliated organizations, or those of the publisher, the editors and the reviewers. Any product that may be evaluated in this article, or claim that may be made by its manufacturer, is not guaranteed or endorsed by the publisher.

## Abbreviations

AF: Atrial fibrillation
BMI: Body mass index
SVD: Cerebral small vessel disease
CHA2DS2-VASc: Congestive heart failure, hypertension, age ≥ 75 years, diabetes mellitus, stroke or transient ischemic attack (TIA), vascular disease, age 65 to 74 years, sex category
MoCA: Montreal cognitive assessment
RAVLT: Rey auditory verbal learning test
STT: Shape trail test
SCWT: Stroop color and word test
BMI: Body mass index
MPRAGE: Magnetization-prepared rapid gradient-echo
WMH: White matter hyperintensities
CMBs: Cerebral microbleeds
EPVS: Enlarged perivascular spaces
ECG: Electrocardiography
DWI: Diffusion-weighted imaging
STRIVE: The STandards for ReportIng Vascular changes on nEuroimaging
MRI: Magnetic resonance imaging
FLAIR: Fluid-attenuated inversion recovery
ADC: Apparent diffusion coefficient
WMH: White matter hyperintensity
EPVS: Enlarged perivascular spaces
BG: Basal ganglia
CSO: Centrum semiovale
