# Supplementary material for: Relationships between cerebral small vessel diseases markers and cognitive performance in stroke-free patients with atrial fibrillation
Source: Front Aging Neurosci. 2023 Jan 4;14:1045910. doi: 10.3389/fnagi.2022.1045910 (PMC9846141; doi:10.3389/fnagi.2022.1045910)
Supplement: Supplementary file 1 [file Data_Sheet_1.docx]

**Supplementary tables**

**Table 1. Univariate analysis between baseline characteristics and cognitive measures in patients with AF**

| **Variables** | **Age** | | **Male** | | **BMI** | | **Persistent/permanent AF** | | **CHA2DS2-VASc score** | |
| --- | --- | --- | --- | --- | --- | --- | --- | --- | --- | --- |
|  | **r** | **p** | **r** | **p** | **r** | **p** | **r** | **p** | **r** | **p** |
| **MMSE** | -0.08 | 0.292 | **0.22** | **0.004** | -0.07 | 0.326 | -0.12 | 0.145 | -0.05 | 0.524 |
| **MoCA-BJ** | -0.04 | 0.631 | **0.28** | **<0.001** | -0.10 | 0.191 | -0.10 | 0.203 | -0.09 | 0.248 |
| **C-RAVLT (IR)** | **-0.18** | **0.033** | 0.13 | 0.108 | 0.11 | 0.203 | -0.01 | 0.846 | -0.09 | 0.241 |
| **C-RAVLT (DR)** | **-0.23** | **0.005** | -0.001 | 0.994 | 0.05 | 0.551 | -0.05 | 0.570 | -0.09 | 0.240 |
| **Stroop-A _time_,s** | **0.18** | **0.029** | -0.12 | 0.158 | -0.12 | 0.152 | -0.08 | 0.328 | 0.06 | 0.459 |
| **Stroop-A _correct_** | -0.11 | -0.187 | 0.14 | 0.094 | 0.04 | 0.610 | 0.07 | 0.423 | -0.04 | 0.600 |
| **Stroop-B _time_,s** | **0.26** | **0.002** | 0.06 | 0.513 | 0.04 | 0.643 | 0.005 | 0.957 | 0.09 | 0.288 |
| **Stroop-B _correct_** | -0.12 | -0.127 | -0.12 | 0.139 | -0.10 | 0.202 | -0.13 | 0.120 | -0.04 | 0.599 |
| **Stroop-C _time_,s** | **0.21** | **0.012** | 0.13 | 0.132 | -0.12 | 0.142 | -0.007 | 0.929 | 0.07 | 0.356 |
| **Stroop-C _correct_** | -0.12 | 0.138 | -0.04 | 0.655 | 0.03 | 0.670 | 0.02 | 0.810 | -0.02 | 0.846 |
| **STT-A,s** | **0.23** | **0.015** | -0.10 | 0.276 | -0.13 | 0.166 | -0.14 | 0.120 | 0.03 | 0.741 |
| **STT-B,s** | **0.34** | **<0.001** | -0.07 | 0.455 | -0.01 | 0.881 | -0.07 | 0.442 | 0.12 | 0.174 |

*Abbreviation：AF: atrial fibrillation; BMI: body mass index; CHA2DS2-VASc (congestive heart failure, hypertension, age ≥ 75 years, diabetes mellitus, stroke or transient ischemic attack (TIA), vascular disease, age 65 to 74 years, sex category); MMSE, Mini-Mental State Examination; MoCA, Montreal Cognitive Assessment-Beijing version; C-RAVLT (IR), The Chinese Rey Auditory Verbal Learning Test(immediate recall); C-RAVLT (DR) , The Chinese Rey Auditory Verbal Learning Test(delayed recall); STT, Shape Trail Test.* *Bold values are statistically significant.*

**Table 2. Univariate analysis between vascular risk factors and cognitive measures in patients with AF**

| **Variables** | Hypertension | | Diabetes mellitus | | Hyperlipidemia | | CHD | | Current smoking | | Current drinking | |
| --- | --- | --- | --- | --- | --- | --- | --- | --- | --- | --- | --- | --- |
|  | **r** | **p** | **r** | **p** | **r** | **p** | **r** | **p** | **r** | **p** | **r** | **p** |
| **MMSE** | 0.05 | 0.517 | 0.03 | 0.623 | -0.09 | 0.283 | 0.05 | 0.571 | 0.12 | 0.140 | 0.14 | 0.088 |
| **MoCA-BJ** | -0.03 | 0.731 | -0.01 | 0.874 | -0.09 | 0.246 | -0.02 | 0.852 | 0.14 | 0.080 | 0.08 | 0.305 |
| **C-RAVLT (IR)** | 0.02 | 0.774 | 0.04 | 0.654 | 0.06 | 0.474 | -0.12 | 0.147 | 0.04 | 0.639 | 0.03 | 0.714 |
| **C-RAVLT (DR)** | 0.001 | 0.994 | 0.015 | 0.854 | 0.02 | 0.832 | -0.09 | 0.269 | -0.002 | 0.979 | 0.02 | 0.792 |
| **Stroop-A _time_,s** | 0.02 | 0.833 | -0.07 | 0.409 | -0.05 | 0.562 | -0.05 | 0.529 | -0.04 | 0.600 | -0.06 | 0.471 |
| **Stroop-A _correct_** | 0.05 | 0.514 | 0.01 | 0.896 | 0.03 | 0.733 | 0.11 | 0.214 | 0.07 | 0.359 | 0.07 | 0.388 |
| **Stroop-B _time_,s** | 0.02 | 0.781 | -0.28 | 0.739 | -0.009 | 0.915 | 0.02 | 0.779 | 0.06 | 0.429 | 0.02 | 0.821 |
| **Stroop-B _correct_** | -0.02 | 0.739 | -0.04 | 0.613 | -0.14 | 0.095 | 0.02 | 0.808 | 0.04 | 0.636 | 0.07 | 0.413 |
| **Stroop-C _time_,s** | 0.03 | 0.646 | 0.05 | 0.489 | -0.06 | 0.498 | 0.06 | 0.513 | 0.08 | 0.307 | -0.03 | 0.709 |
| **Stroop-C _correct_** | 0.09 | 0.290 | -0.04 | 0.653 | -0.09 | 0.254 | 0.06 | 0.515 | -0.02 | 0.836 | -0.01 | 0.901 |
| **STT-A,s** | -0.12 | 0.211 | 0.03 | 0.752 | -0.10 | 0.333 | -0.11 | 0.225 | -0.16 | 0.090 | -0.03 | 0.777 |
| **STT-B,s** | **-0.19** | **0.042** | -0.008 | 0.931 | -0.09 | 0.359 | -0.04 | 0.706 | -0.17 | 0.065 | -0.16 | 0.076 |

*Abbreviation: CHD,* Coronary heart disease; *MMSE, Mini-Mental State Examination; MoCA, Montreal Cognitive Assessment-Beijing version; C-RAVLT (IR), The Chinese Rey Auditory Verbal Learning Test(immediate recall); C-RAVLT (DR), The Chinese Rey Auditory Verbal Learning Test(delayed recall); STT, Shape Trail Test. Bold values are statistically significant.*
